# Posıtıve effect of platelet rich fibrin on osseointegration

**DOI:** 10.4317/medoral.21026

**Published:** 2016-07-31

**Authors:** Elif Öncü, Burak Bayram, Alpdoğan Kantarcı, Serap Gülsever, Emine-Elif Alaaddinoğlu

**Affiliations:** 1 Necmettin Erbakan University, Departmant of Periodontolgy, Konya, Turkey; 2 Baskent University, Departmant of Oral and Maxillofacial Surgery, Ankara, Turkey; 3 Department of Applied Oral Sciences, Center for Periodontology, The Forsyth Institute, Cambridge, MA, USA; 4 Baskent University, Departmant of Periodontolgy, Ankara, Turkey

## Abstract

**Background:**

Leukocyte-platelet rich fibrin (L-PRF) is a second generation platelet concentrate clinically used to accelerate tissue healing and bone regeneration. Achieving reduced implant osseointegration time could provide immediate or early loading of implants. The aim of this study was to evaluate the L-PRF-induced osseointegration and bone-implant contact (BIC) in an experimental animal model.

**Material and Methods:**

Twelve 4-month-old New Zealand white rabbits were used. Following general anesthesia, 3-5 mL of blood was obtained from the central artery in rabbit ear and L-PRF was prepared. Two implant cavities (5 mm long and 3 mm in diameter) were created in each tibia with a total of four cavities in each animal. Two of these cavities were selected and covered with PRF (test group). The remaining L-PRF was used to soak the implants placed into the L-PRF covered sockets. Other cavities were left as controls. In total, 48 implants were placed. Animals were sacrificed after two, three, or four weeks. Histological samples were obtained and peri-implant tissues were histomorphometrically evaluated for bone-to-implant contact and new bone formation.

**Results:**

Histomorphometric analyses of the defects revealed that the L-PRF was detectable up to the second week. Application of L-PRF increased the rate and amount of new bone formation in the experimental group compared to the control group. Bone-to-implant contact was enhanced when the surface was pre-wetted with L-PRF (*p*<0.01).

**Conclusions:**

The results of this study demonstrated that L-PRF application may increases amount and rate of new bone formation during the early healing period and provides a faster osseointegration around implants.

**Key words:**Dental implants, platelet rich fibrin, osseointegration, bone regeneration, matrix for growth factors.

## Introduction

Osseointegration of dental implants is critical for the long-term success and stability. Various strategies have been used to accelerate the time required for osseointegration without compromising the mechanical outcomes and tissue integration (1,2). The implant surface topography plays a key role in the early stages of bone-to-implant contact (BIC); peri-implant bone formation depends on the healing capacity of the bone (2,3). Modification of the surface properties of the implants by chemical methods such as incorporation of inorganic phases on or into the titanium oxide layer and physical enhancement of the materials by increasing the level of roughness have successfully increased the BIC (3). Another strategy to reduce the osseointegration time has been the modulation of the healing response after the implant placement (2,3). This has been accomplished by biologically active molecules during implant placement to induce osteoconductivity, increase osteoblastic differentiation and enhance healing of peri-implant bone (3,4). Growth factors, bone-specific proteins, bone morphogenetic proteins induce better osseointegration through replication and differentiation of osteoprogenitor cells and interfacial tissue maturation (3-8).

Platelet-based preparations from patient’s own blood provide an inexpensive alternative to commercially available bioactive materials. Activated platelets secrete a wide range of proteins and growth factors including, Bone Morphogenetic Protein (BMP), Platelet-Derived Growth factor (PDGF), Insulin - like Growth Factor (IGF), Vascular Endothelial Growth Factor (VEGF), Transforming Growth Factor-β1 (TGF-β1) and Transforming Growth Factor-β2 (TGF-β2), which play key roles in bone healing (5-9). They attract undifferentiated mesenchymal cells to the injured site and facilitate angiogenesis, chemotaxis and cell proliferation. Growth factors also control the synthesis and degradation of extracellular matrix proteins, enhance osteogenesis and potentially accelerate peri-implant wound healing and osteointegration (8-11).

Leukocyte-Platelet rich fibrin (L-PRF) is a second generation of autologous platelet concentration and a fibrin mesh consisting of leukocytes, growth factors, proteins and cytokines (9). L-PRF, platelet rich plasma (PRP) and platelet rich growth factor (PRGF) are structurally different materials. L-PRF has advantages over PRP and PRGF by having a strong fibrin structure and not requiring any biochemical modification through bovine thrombin or anticoagulants (12,13). L-PRF has a very significant slow sustained release of key growth factors for at least 1 week and up to 28 days, which stimulates its environment for a significant time during early phases of wound healing (10-14). Because of its natural fibrin framework properties, growth factors can keep their activity for a relatively longer period and promote tissue regeneration (15). When L-PRF was applied to the titanium implant surfaces, growth factors covered the implants forming a fibrin layer for platelets to adhere (5-8,15). Recently evaluated the effect of PRP and PRF on proliferation and differentiation of rat osteoblasts. This study showed that PRF released autologous growth factors gradually and expressed stronger and more durable effect on proliferation and differentiation of rat osteoblasts than PRP *in vitro* (14,15).

Yet, *in vivo* justification for L-PRF application during implant placement and its impact on osseointegration is limited. Therefore, we have hypothesized that implementation L-PRF around dental implants may lead to faster healing rates in peri-implant bone and decrease osseointegration time. In order to test this hypothesis, we have used a rabbit model and studied the effects of L-PRF on new bone formation and BIC around dental implants.

## Material and Methods

- Animal Model, Preparation of the L-PRF and Surgical Procedure

**Figure 1:**
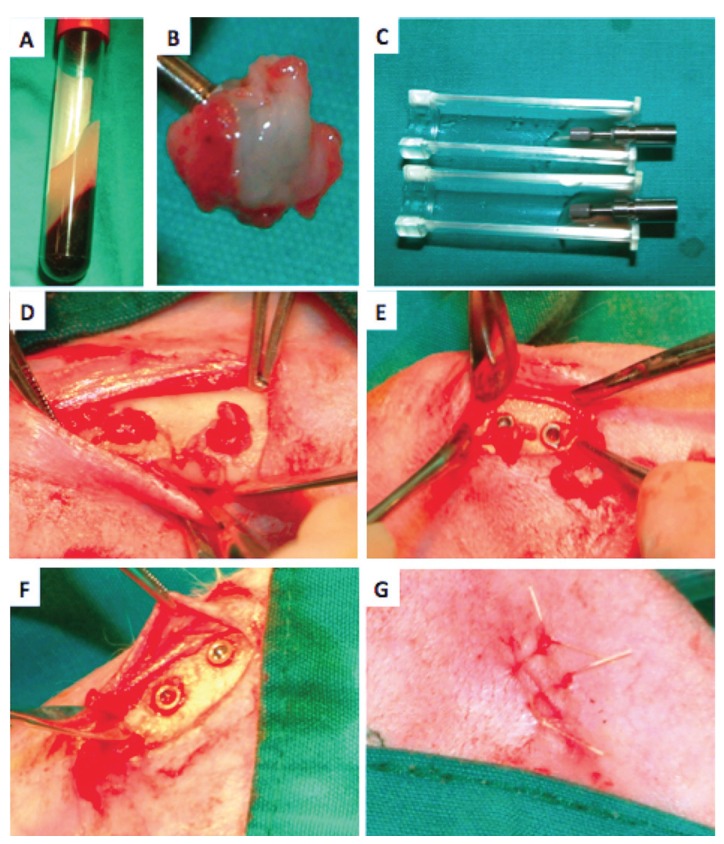
Study design and sequence of events. Panel A. L-PRF clot in the middle of the tube, Panel B. Fibrin clots transferred to L-PRF Box, Panel C. The implants were washed completely, Panel D. L-PRF membrane placed in implant socket, Panel E. Implants placed in sockets which coated with L-PRF, Panel F. Implants placed in sockets in control group, Panel G. The periosteum and skin were closed in a layer using 5-0 vicryl resorbable sutures.

The research protocol was submitted to and approved by the Institutional Animal Care and Use Committee and ethical board of animal investigations. Twelve 4-month old New Zealand white rabbits with an average weight of 3.0-3.5 kg were used. Each rabbit was individually caged and fed. General anesthesia was induced by intramuscular injection of a combination of 0.5 mL ketamine (20 mg/kg by body weight; Ketalar, Eczacıbası, Istanbul, Turkey) and 0.5 mL xylazine (10 mg/kg by body weight; Rompun, Bayer, Leverkusen, Germany). After general anesthesia, 3 to 5 mL of blood was obtained from the central artery of the ear. Samples were collected in 9 mL glass-coated plastic tubes without anti-clotting agent (Becton Dickinson Vacutainer, Franklin Lakes, NJ, USA) and immediately centrifuged at 2700 rpm for 12 minutes with a table centrifuge (PC-02, Process Ltd., Nice, France; Fig. 1A). The leukocyte-rich fibrin clot formed in the middle part of the tube was taken and remnants of red blood cells were scraped off with gauze. The clot was then transferred to the PRF box (Process Ltd., Nice, France), compressed and PRF membranes were obtained (Fig. 1B). Serum obtained during compression of the fibrin clot was transferred to a syringe.

Distal surfaces of right and left tibias were shaved and disinfected with a povidine-iodine solution. Local anesthesia was accomplished (Ultracaine D-S®, Hoechst A.G, Turkey). Surgical procedures were performed by the same surgeon (E.Ö.). After a crestal incision, the muscles were dissected and a sharp subperiosteal dissection was used to reflect the periosteum to expose the tibia bone. Two implant site preparation, which were 5-mm apart from each other, were prepared. Mini implants specially produced for application in rabbit tibia were used (SLA surface; Nucleoss, Izmir, Turkey). Two implants in left tibia were used as “test” and other two implants in the right tibia of the same animal were assigned as “control”. A total of 48 implants (3 mm width and 5 mm length) were used. L-PRF membranes were applied to test sockets (Fig. 1C). Test implants were thoroughly soaked with L-PRF before implant insertion (Fig. 1D) and placed (Fig. 1E). Control implants were placed without the PRF application (Fig. 1F). The periosteum and skin were closed in layers using 5-0 resorbable sutures (Fig. 1G).

**Figure 2:**
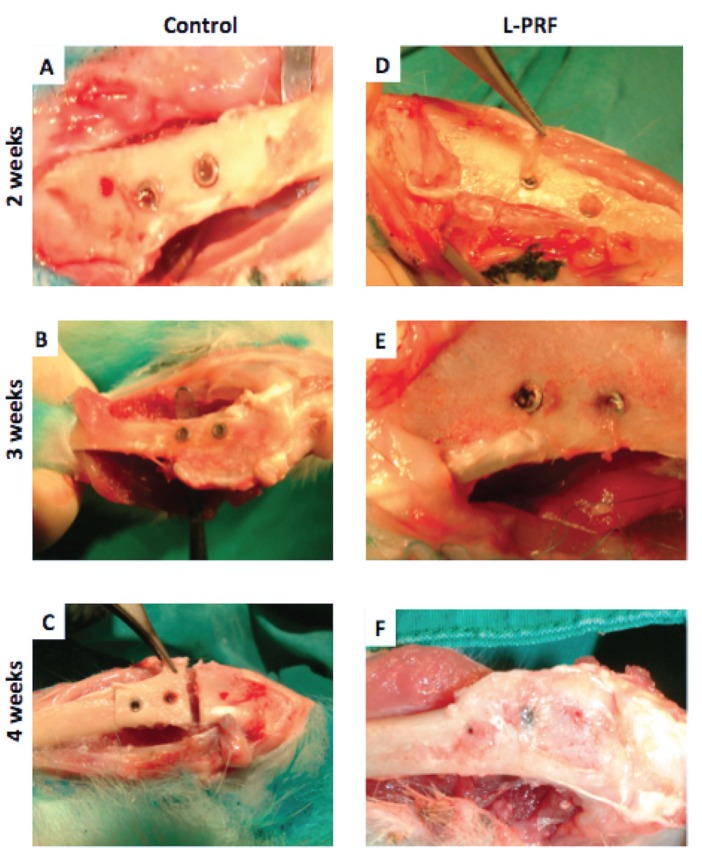
Second, third and fourth weeks macroscopic view of the test and control groupsafter sacrifice. Panel A. The image of the control group at 2nd week, Panel B. The image of the control group at 3rdweek, Panel C.The image of the control group at 4th week, Panel D.The image of the test group at 2nd week, L-PRF was not resolved in the second week of the test groups was shown by the red arrow, Panel E. The image of the test group at 3rd week, implants uppermost coated with bone tissue, Panel F. The image of the test group at 4th week, implants cannot be observed due to new bone formation at the peak of the implants.

All animals were postoperatively medicated with intramuscular amoxicillin (1.5 mg/kg body weight) for infection control and buprenorphine (10 mg) for pain relief. Animals were sacrificed on 2nd, 3rd and 4th weeks post-surgery and histological samples were obtained (Fig. 2).

- Histological Analyses 

Samples were ﬁxed in neutral buffered formalin, dehydrated in 70%, 90%, 95% and 100% alcohol and embedded in a blue light-curing resin (Technovit 7200 VLC; Kulzer, Wehrheim, Germany) for 48 hours. After the dehydration, each specimen was embedded in methylmethacrylate. Samples were then sectioned along the long axis of the implant using high-speed rotational microtome (Micromet, Remet, Italy). Finally, the sections were ground to a thickness of 40-µm and then were stained with 1% toluidine blue. Histological examination was made with Zeiss Axiovert 200 microscope (Carl Zeiss inverted microscope for transmitted light and epifluorescence, Germany), which was connected to a computer and photomicrographs were taken with a under UV light with a same microscope. Before performing image analysis, a specific region of interest (ROI) had been determined. The proportion of new bone formation related areas of bone detected inside the ROI divided by the total surface area and analyzed the effect of L-PRF on bone formation, the newly formed bone on the surface of the implant and BIC were evaluated. The total amount of new bone was calculated according to percentage of the total region on the surface of the implant. The bone-to-implant contact was calculated by the percentage of the bony contact to the implant surface.

- Statistical Analysis 

Statistical computations were carried out using IBM PASW/SPSS software (v.18.0.0 2009, IBM Corporation, Somers, NY, USA). Implants were included in the statistical analysis as independent values. Mean values and standard deviations were calculated for each variable and group. The difference between groups was analyzed with ANOVA and the difference within groups was analyzed with Student t-test.

## Results

All implants successfully healed without any postoperative complications. No mortality, infection or wound exposure or change in body weight was noted.

**Figure 3:**
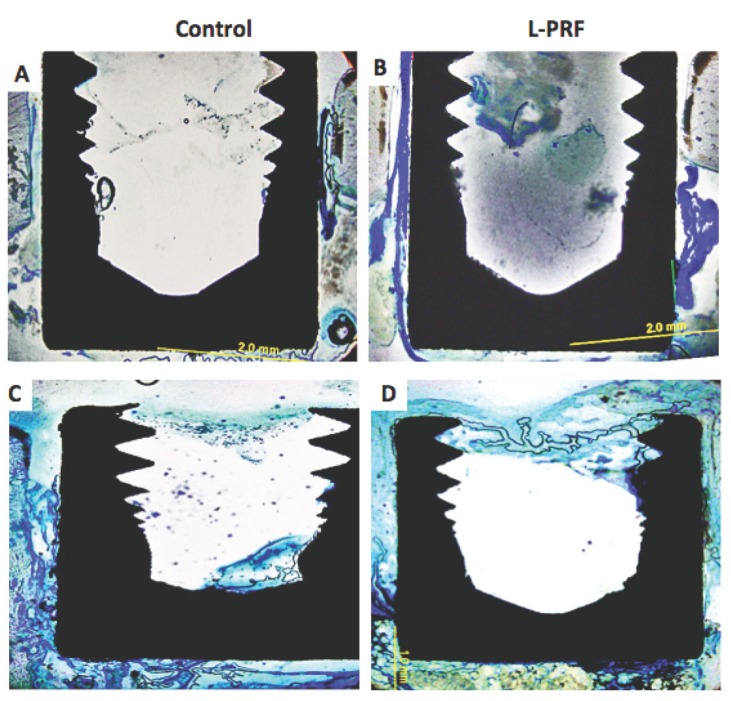
Photographs of the histological sections seen by light microscopy at second and fourth week view of the image in test and control groups. Sections were stained with toluidine blue. Original magniﬁcation, 20×. Panel A.Photographs of the histological sections seen by light microscopy at 2nd weeks in control group, percentage of new bone formation and bone to implant contact was shown. Panel B Photographs of the histological sections seen by light microscopy at 2nd weeks in test group, L-PRF was not resolved in the second week of the test groups was shown. Panel C. Photographs of the histological sections seen by light microscopy at 4th weeks in control group, percentage of new bone formation and bone to implant contact was seen, there was almost no bone contact in the uppermost on the implant surface . Panel D. Photographs of the histological sections seen by light microscopy at 4th weeks in test group, the regenerated bone covered nearly all the surface in this group.

Histomorphometric analyses of the defects revealed that the L-PRF was detectable up to the second week (Fig. 2D,3B). In the test group, L-PRF around implants was obvious on the third and fourth weeks with a substantial new bone formation. At the end of 4 weeks, new bone formation as stained with toluidine blue around implant was limited with almost no bone contact on the implant surface in the control group (Fig. 3C). Widely stained bone formation and almost complete healing around implants were observed in the L-PRF group (Fig. 3D). Application of L-PRF increased the rate and amount of new bone formation in the experimental group compared to the control group (31.7% ± 6.8% vs 12.1% ± 7.7% at third week and 39.9% ± 3.9% vs 26.4% ± 9.0% at fourth week, *p*<0.01).

**Figure 4:**
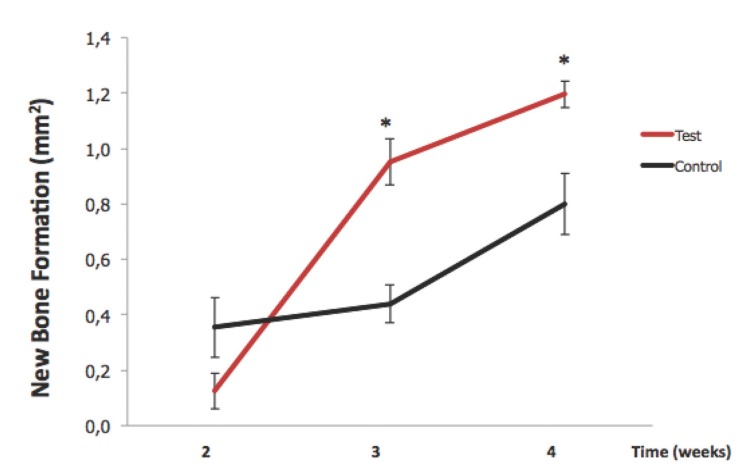
Percentage of new bone formation in the test and control groups.

There was a rapid increase in the percentage of new bone formation and BIC in the test group (Fig. 4). Bone-to-implant contact was enhanced when the surface was pre-wetted with L-PRF (52.6% ± 21.7% vs 36.0% ± 23.2% at third week and 54.6% ± 5.2% vs 39.0% ± 8.9% at fourth week *p*<0.01).

There was no evidence for ﬁbrotic tissue layer formation in any groups. The percentage of new bone formation around the implants in the test group was signiﬁcantly higher than the control group (*P* < 0.01). Likewise, the BIC was signiﬁcantly higher in the test group than the control group on the third and fourth weeks (*P* < 0.01).

## Discussion

We have investigated the potential effects of L-PRF on stimulating bone regeneration and accelerating the osseointegration of dental implants. The histomorphometric results showed the rate of bone formation and BIC were enhanced in the L-PRF treated sockets compared to the empty sockets after the implant placement.

In this study mean bone-to-implant contact in the 4th week was 54.61% in the experimental group and 26.44% in the control group. The findings of this study were in agreement with the results reported by Anitua *et al.* Using a similar approach, these authors reported a 51% BIC when implants were coated with PRGF compared to the 22% BIC of the control group after 8 weeks of healing time (5-8). Our results also confirm the data presented by Lee *et al.* who have reported that the mean new bone formation was 29% in the experimental group and 11% in the control group with a BIC of 39% vs 17% (16). Furthermore, our results support the findings by Fontana *et al.* and those of another study where PRP improved BIC for roughened implants inserted in rat tibias (11,15). Using a similar approach, Fuerst *et al.* reported a 55% BIC when implants were coated with PRGF versus 39% of the control group after 4 weeks of healing time (17).

In another rabbit study, histomorphometric results showed that during the first 2-3 weeks, integration of titanium mini-screws and bones was weak but after 4 weeks of healing integration of titanium mini-screws and bones was significantly stronger. They stated that four weeks is a critical time point during the progress of integration of titanium mini-screws and bones (18). In this study there is virtually no difference in BIC in the control group between 2, 3 and 4 weeks, these results may have been due to the lack of sufficient time for osseointegration in the control groups. Histomorphometric results showed BIC in theexperimental group than in the control in the 3rd and 4th weeks. Differences between groups may be from the fact that either follow-up period is short for osseointegration, or PRF speeds up the healing.

Some researchers argue that the growth factors released by platelets play an important role in enhancing the bone response and in the transformation of marrow bone cells into osteoblasts (7,10-15). Other studies have also demonstrated the potential effects of different platelet rich products in accelerating the regeneration of bone tissues. It is thought that the implant surfaces activate the platelets leading to a rapid osteointegration (5,8,19). Platelet Rich Fibrin is a recognized support matrix for BMP transplants. Therefore, the fibrin matrix associated with BMPs has angiotrophic, hemostatic, and osseous conductive properties (10,20). L-PRF may improve and accelerate osteogenesis (10). Previously, PDGF and PRP were applied around implants in order to provide the regeneration of bone and increase BIC in many studies (7-10). We preferred L-PRF, because L-PRF, PRP and PRGF are structurally different materials. The advantages of PRF technique over PRP and PRGF include shorter time of preparation, lack of requiring anticoagulant and bovine thrombin. In course of the platelet and fibrinogen activation of PRGF and PRP growth factors and some other proteins are not enmeshed in the fibrin network, because the fibrin polymerization is incomplete. These molecules are therefore released quickly during the first hours after preparation. Furthermore these products do not contain leukocytes and cannot sustain the production of new growth factors after the initial release. On the other hand, the strong fibrin architecture of the L-PRF allows an intense slow release throughout the entire span of the experiment, and the release is further supported by the production of new growth factors by the leukocytes living in the L-PRF membrane (4,11,17,18,21). When L-PRF applied to the implant surface, a protein layer involving massive growth factors are constituted. This regeneration potential may stimulate the healing of implant on the surrounding bone with the platelet rich layer (4).

To our knowledge, the present study is the first experimental study, which evaluated the effect of L-PRF on osseointegration. Within the limits of our study, we concluded that L-PRF application during implant placement may increases the rate and enhances the osseointegration providing a convenient and affordable choice for implant placement especially in sites where earlier loading may be required. Clinical studies are needed to demonstrate efficacy in humans.
